# Obituary

**Published:** 1910-03-19

**Authors:** 


### Obituary.

Mr. Alfred John Wright, M.R.C.S., L.S.A., has died at Markfield, Leicester, at the age of 47. He took the above degrees in 1886, and was a student at Charing Cross Hospital. He became District Medical Officer and Public Vaccinator to the Market Bosworth Union, and was a certificated factory surgeon.

The death is announced of Mr. George Annesley Derville Mahon, J.P., suddenly, at the age of 68. Mr. Mahon took his M.R.C.S. and L.S.A. in the year 1862, and was a student of St Mary's Hospital, where later he held the posts of registrar, house surgeon, and resident accoucheur. He had been also resident surgeon at the Lock Hospital, London, and was a J.P. for Bedfordshire.

The death has taken place at Chester of Mr. George Arthur Kenyon, at the age of 66. He was a M.B. of London (1868), and had taken his diploma two years previously. For thirty-two years he was medical officer of health to Chester, and his appointments included that of house surgeon to Hospital for Women and resident obstetric assistant to St. George's Hospital. His principal publication was the "Chester Health Reports," 1824-1904.

Surgeon-Major-General George Langford Hinde, C.B., R.A.M.C., has died at Reading at the age of seventy-seven. He entered the Army in 1855 and served in the Crimea, was present at the siege of Sebastopol, and throughout the Transvaal campaign in 1881, and for his services in the Sudan Expedition in 1884-5 he obtained the medal with clasp and the bronze star. He became a surgeon-major-general in 1892, when he retired from the Service. In 1885 he became an L.R.C.S. of Ireland.

